# The Tetragonal Monoxide of Platinum: A New Platform for Investigating Nodal-Line and Nodal-Point Semimetallic Behavior

**DOI:** 10.3389/fchem.2020.00704

**Published:** 2020-08-14

**Authors:** Yang Li, Jihong Xia, Vipul Srivastava

**Affiliations:** ^1^Faculty of Mechanical and Electrical Engineering, Kunming University of Science and Technology, Kunming, China; ^2^Department of Physics, Chongqing University of Arts and Sciences, Chongqing, China; ^3^Department of Physics, School of Chemical Engineering and Physical Sciences, Lovely Professional University, Phagwara, India

**Keywords:** double nodal lines, fermi arc and drum-head-like surface states, triple point, DFT, phonon dispersion, mechanical behaviors

## Abstract

The search for new topological materials that are realistic to synthesize has attracted increasing attention. In this study, we systematically investigated the electronic, mechanical, and topological semimetallic properties, as well as the interesting surface states, of the tetragonal monoxide of platinum, which is realistic to synthesize, via a first-principles approach. Our calculated results indicate that PtO is a novel topological semimetal with double nodal lines in the *k*_*z*_ = 0 plane and a pair of triple topological nodal points along the A'-M-A directions. Obvious surface states, including Fermi arc and drum-head-like surfaces, could be found around nodal points and nodal lines. The dynamic and mechanical stabilities of *P4*_2_*/mmc*-type PtO were examined in detail via calculation of the phonon dispersion and determination of elastic constants, respectively. Some other mechanical properties, including the bulk modulus, Young's modulus, shear modulus, Poisson's ratio, and Pugh's index, were considered in this study. *P4*_2_*/mmc*-type PtO provides a good research platform for investigation of novel behaviors that combine mechanical properties and rich topological elements.

## Introduction

As rising stars in the topological material family, topological semimetals (Fang et al., 2012, 2016; Chiu and Schnyder, 2014; Yan and Felser, 2017; Gao et al., 2019), whose band crossing points form 0-D nodal point, 1-D nodal line, or 2-D nodal surface states in momentum space, have recently attracted extensive attention. Topological nodal point semimetals (Hosur et al., 2012; Zyuzin and Burkov, 2012; Hosur and Qi, 2013; Vazifeh and Franz, 2013; Liu et al., 2014; Lundgren et al., 2014; Kobayashi and Sato, 2015; Miransky and Shovkovy, 2015; Xu et al., 2015a; Young and Kane, 2015) enjoy 0-D nodal points in momentum space. Topological nodal line semimetals (Cai et al., 2018; Chen et al., 2018; Gao et al., 2018; Zhou et al., 2018; He et al., 2019; Jin et al., 2019a; Pham et al., 2019; Yi et al., 2019; Zou et al., 2019; Zhao et al., 2020) host 1-D topological nodal lines in momentum space via band crossing along a line in momentum space. Topological nodal surface semimetals (Wu et al., 2018; Zhang et al., 2018; Fu et al., 2019a; Qie et al., 2019; Yang et al., 2020) host 2-D nodal surface states that are composed of continuous band crossing points.

In addition, topological semimetals exhibit many types of band crossing points based on degeneracy. Weyl and Dirac semimetals feature 2-fold and 4-fold degenerate 0-D band crossing points, respectively. In detail, Weyl semimetals (Hosur et al., 2012; Zyuzin and Burkov, 2012; Hosur and Qi, 2013; Vazifeh and Franz, 2013; Lundgren et al., 2014; Yan and Felser, 2017) have nodal points that are protected by inversion (*P*) or time-reversal symmetries (*T*). Dirac semimetals (Liu et al., 2014; Lundgren et al., 2014; Kobayashi and Sato, 2015; Miransky and Shovkovy, 2015; Young and Kane, 2015) host quadruple degenerate nodal points that are protected by crystalline symmetry. Furthermore, topological semimetals with 3-, 6-, and 8-fold degenerate band crossing points have been considered (Weng et al., 2016a,b; Cai et al., 2018; Kumar et al., 2020) by researchers. Of these, triply degenerate nodal point-type semimetals (Weng et al., 2016a,b) are well-studied due to their novel topological elements and related surface states. Triple nodal points can appear both in isolation and at nodal line connections.

Many topological semimetals that are realistic to synthesize and have various types of band crossing points have been proposed. Unfortunately, these band crossing points are usually disturbed by other trivial bands near the Fermi level, which covers novel physics behaviors from the band crossing points. Hence, to search for topological behaviors of topological semimetals with rich topological elements, it is necessary to find topological semimetals with clean band structures near the Fermi level. Thus far, there are few potential clean topological semimetals with more than one topological element. This greatly impedes further investigation of realistic topological semimetals with rich topological elements.

In this work, we focus on an old realistic material, tetragonal PtO with ICSD IDs^1^ 164290 and 26599. In 1941, Moore Jr and Pauling (Moore Pauling, 1941) synthesized PdO and PtO by the method of Shriner and Adams, involving fusing palladous chloride and potassium nitrate, and platinous oxide by a similar method. Based on previous powder photographic X-ray data, the tetragonal crystal PtO (Moore Pauling, 1941) exhibits a *P4*_2_*/mmc* type structure with lattice constants *a* = *b* = 3.04 ± 0.03 and *c* = 5.34 ± 0.05Ǻ. In this paper, we use a first-principles analysis to investigate its electronic and mechanical properties, as well as its phase stability, systematically. We report its interesting 0-D and 1-D topological elements and the related surface states.

## Computational Details

First-principles calculations were performed using the Vienna *ab initio* simulation package (VASP) (Hafner, 2007) with density functional theory (DFT) (Lejaeghere et al., 2016). The generalized gradient approximation (GGA) (Perdew et al., 1996) of the Perdew–Burke–Ernzerhof (PBE) functional (Ernzerhof and Scuseria, 1999) was selected for the exchange-correlation potential. The projector augmented wave (PAW) (Kresse and Joubert, 1999) pseudo-potential was employed with a cutoff energy of 600 eV for plane-wave expansions. The energy and force convergence criteria were set to 10^−6^ eV per atom and 0.0005 eV/Ǻ, respectively. The surface states were obtained using the Wannier-tools package (Villanova and Park, 2018). The phonon dispersion of 2 × 2 × 2 supercell of PtO monolayer was checked based on density functional perturbation theory (DFPT).

## Results and Discussion

### Structural Model and Dynamic Stability

**Figure 1:**
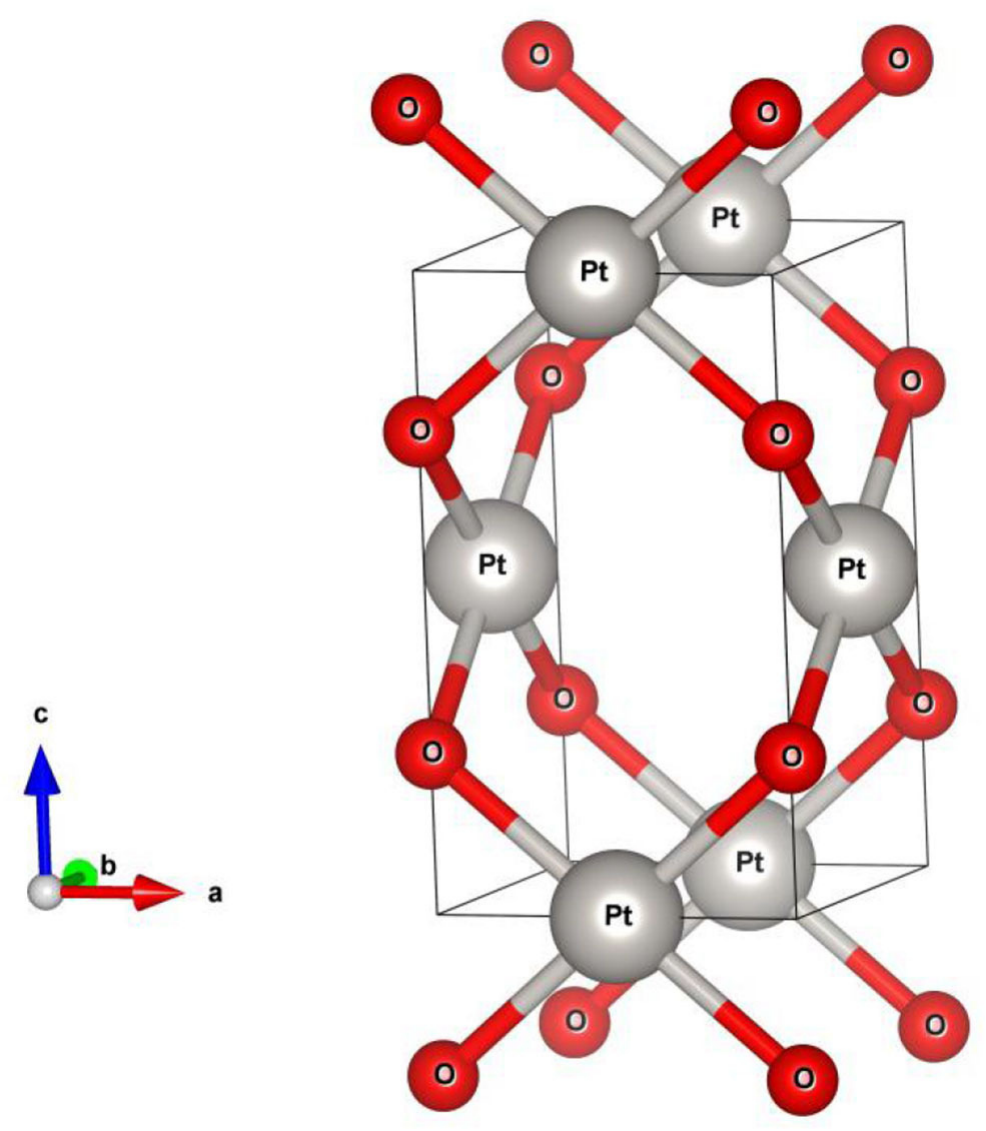
Crystal model of tetragonal PtO.

PtO (Moore Pauling, 1941) crystallizes in a tetragonal structure (as shown in Figure 1) with space group *P4*_2_*/mmc* (no. 131). The minimum energy approach was used for structural optimization. The PtO primitive cell contains two O and two Pt atoms. The atomic positions and equilibrium lattice constants were determined after complete relaxation. The resulting lattice constants are *a* = *b* = 3.15 and *c* = 5.37Ǻ, and are in a good agreement with the experimental data. In their relaxed atomic positions, the Pt and O atoms occupy the 2c (0.0, 0.5, 0.0) and 2e (0.0, 0.0, 0.25) Wyckoff sites, respectively. We would like to point out that the results of current study will retain if the experimental lattice constants are selected, as shown in Figure S1.

**Figure 2:**
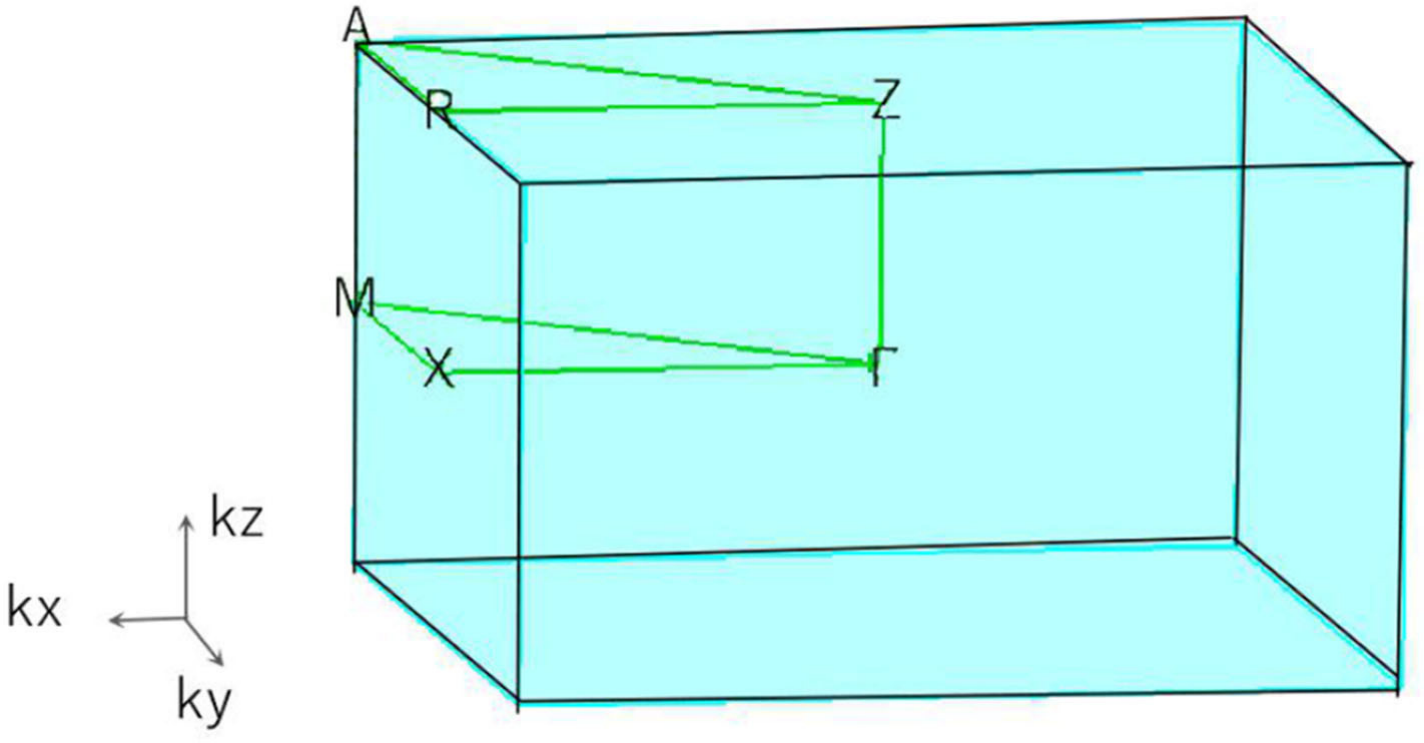
The 3-D bulk Brillouin zone (BZ) of tetragonal PtO.

**Figure 3:**
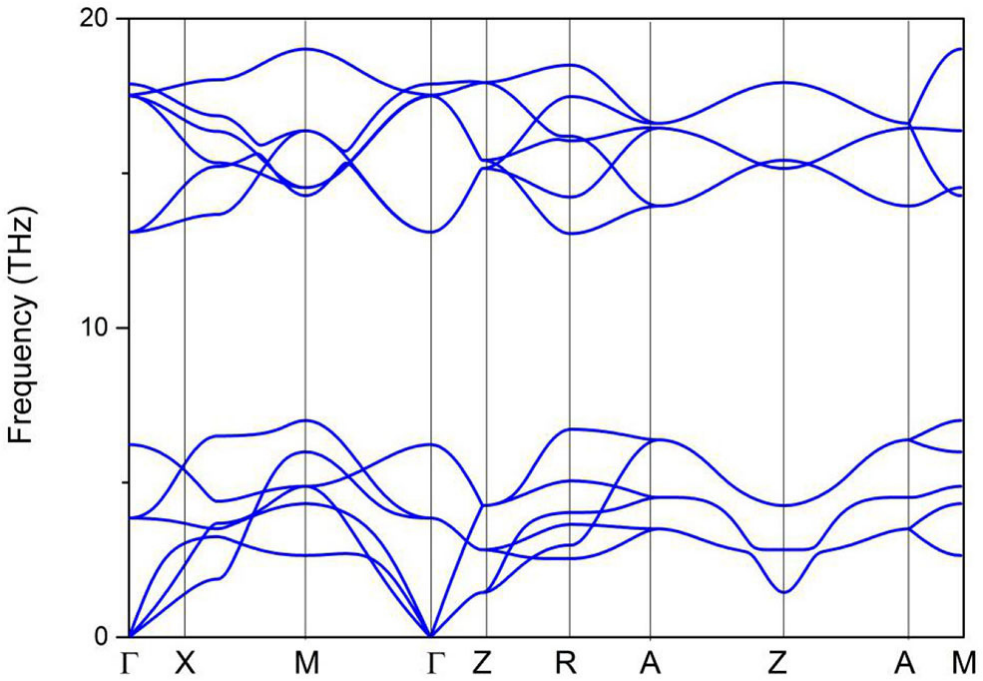
Phonon dispersion of tetragonal PtO along the Γ-X-M-Γ-Z-R-A-Z-A-M directions.

Based on the 3-D bulk Brillouin zone (BZ) selected in Figure 2, the phonon dispersion (Sultana et al., 2018; Abutalib, 2019; Ding et al., 2019; Fu et al., 2019b; Han et al., 2019; Jia et al., 2019) was determined in order to examine the dynamic stability of tetragonal PtO. It is well-known that materials are dynamically stable when no imaginary phonon modes exist in their phonon dispersion curves. Figure 3 shows the calculated phonon dispersion along the Γ-X-M-Γ-Z-R-A-Z-A-M directions. Since only positive frequencies appear in Figure 3, PtO is a dynamically stable material.

### Mechanical Properties and Mechanical Stability

By analyzing the elastic constants, we can obtain information about the mechanical stability of PtO. In this paper, we use the energy-strain method to compute six independent elastic constants. The results are shown in Table 1.

**Table 1:** Calculated PtO elastic constants.

***C_**11**_* (GPa)**	***C_**12**_* (GPa)**	***C_**13**_* (GPa)**	***C_**33**_* (GPa)**	***C_**44**_* (GPa)**	***C_**66**_* (GPa)**
232.61	130.02	169.23	315.41	22.83	38.40

Tetragonal PtO has six independent elastic constants, *C*_11_, *C*_12_, *C*_13_, *C*_33_, *C*_44_, and *C*_66_. We can use the Born–Huang criteria (see criteria i, ii, and iii) to test the mechanical stability of tetragonal PtO:

Criteria (i) *C*_11_ > |*C*_12_|;
Criteria (ii) 2*C*_13_^2^<*C*_33_(*C*_11_ + *C*_12_); and
Criteria (iii) *C*_44_ > 0.

The Born–Huang criteria indicate that PtO is mechanically stable. Other useful mechanical parameters, including the bulk modulus (*B*), shear modulus (*G*), Young's modulus (*E*), Poisson's ratio (*v*), and Pugh's index (*B/G*) are shown in Table 2.

**Table 2:** Elastic behaviors of tetragonal crystal PtO.

***B***	***G***	***E***	***v***	***B/G***
185.62	38.83	108.90	0.402	4.780

**Figure 4:**
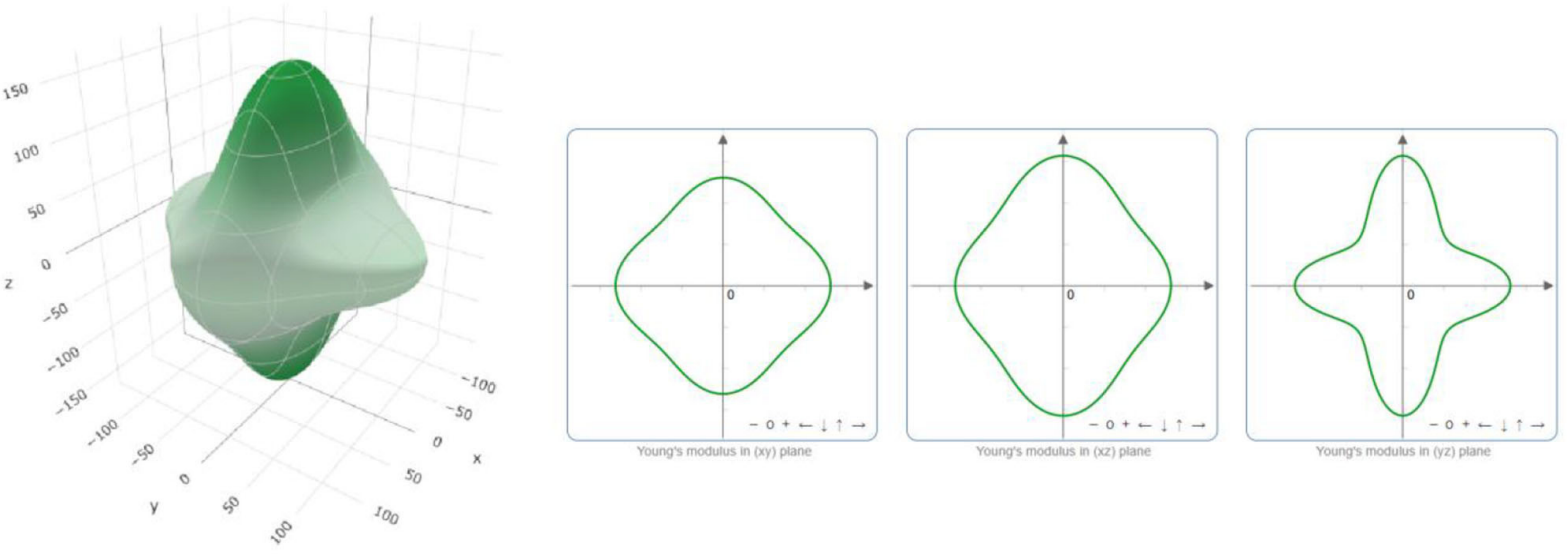
Directional dependence of the Young's modulus.

**Figure 5:**
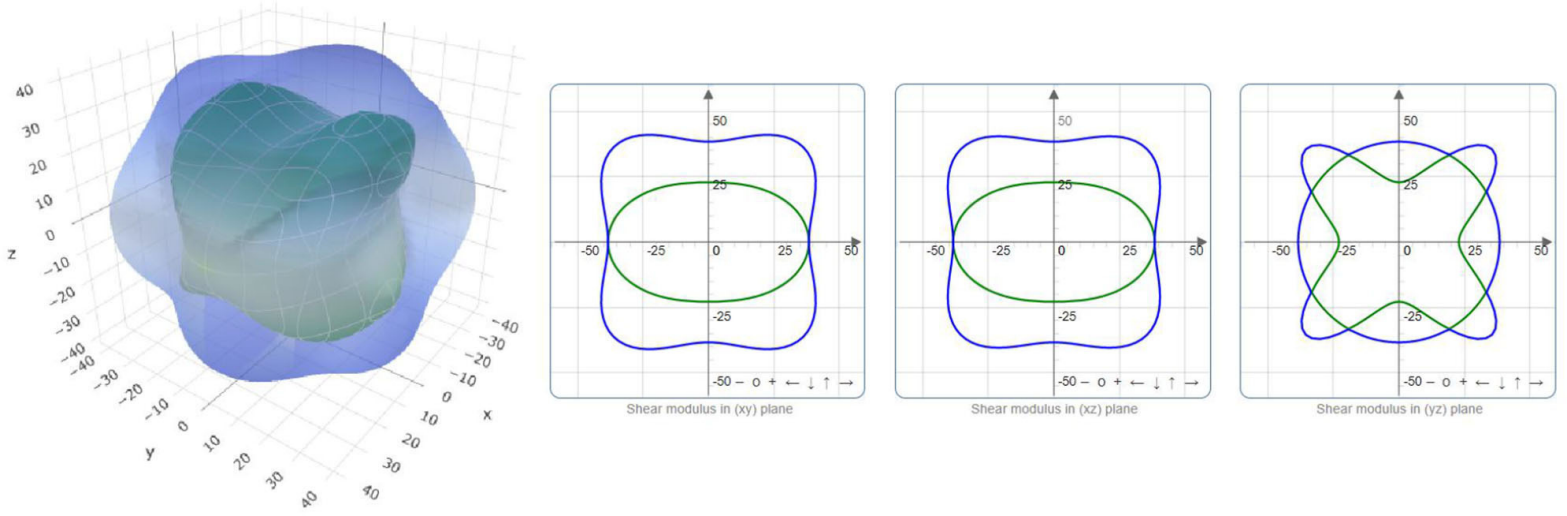
Directional dependence of the shear modulus: blue and green indicate the maximum and minimum values, respectively.

**Figure 6:**
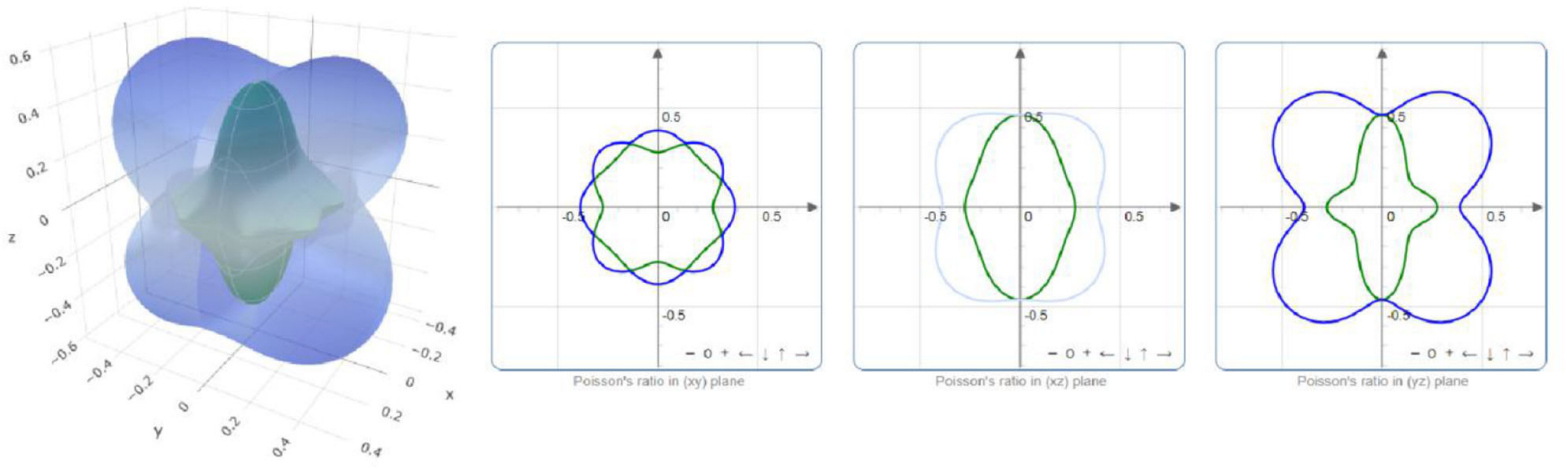
Directional dependence of Poisson's ratio: blue and green indicate the maximum and minimum values, respectively.

The critical value *B/G* that is used to distinguish between brittle and ductile crystals is 1.75. Obviously, PtO is in hand elastically ductile. Moreover, the critical value of *v* that distinguishes between the ionic and covalent chemical band natures is ~0.25. The chemical bonds in a PtO tetragonal crystal are mainly ionic. We use the ELATE program to determine the directional dependence anisotropy of PtO on the Young's modulus, shear modulus, and Possion's ratio in Figures 4–6, respectively. The elastic anisotropy of PtO can be determined from these figures. This is quite important for future practical applications of this material.

### Topological Elements and Novel Surface States

**Figure 7:**
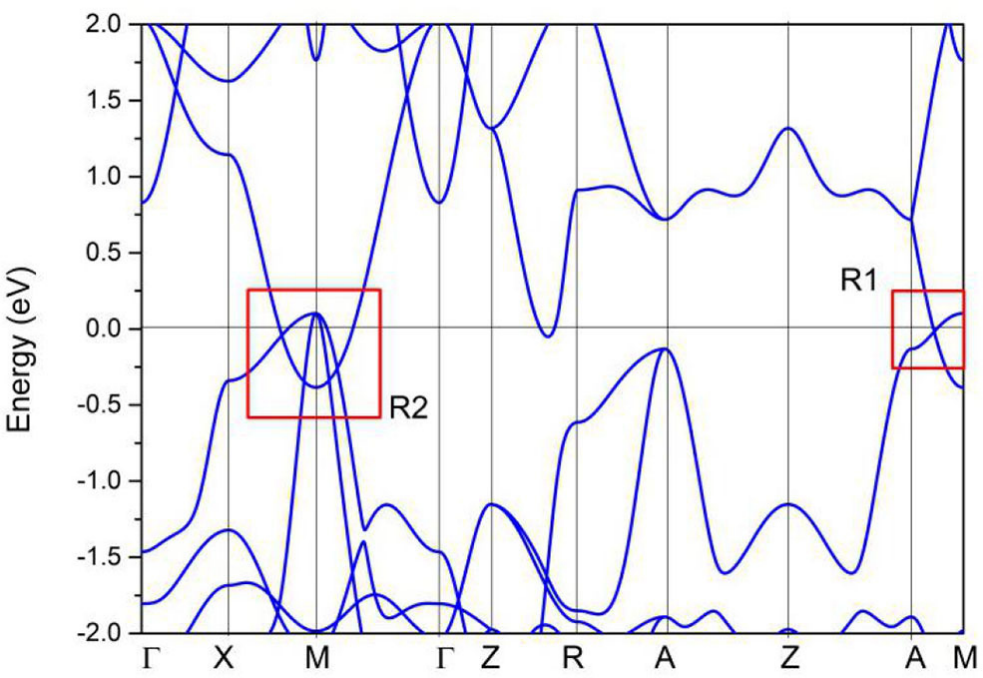
Calculated band structure of PtO along the Γ-X-M-Γ-Z-R-A-Z-A-M directions, as determined using the GGA method.

**Figure 8:**
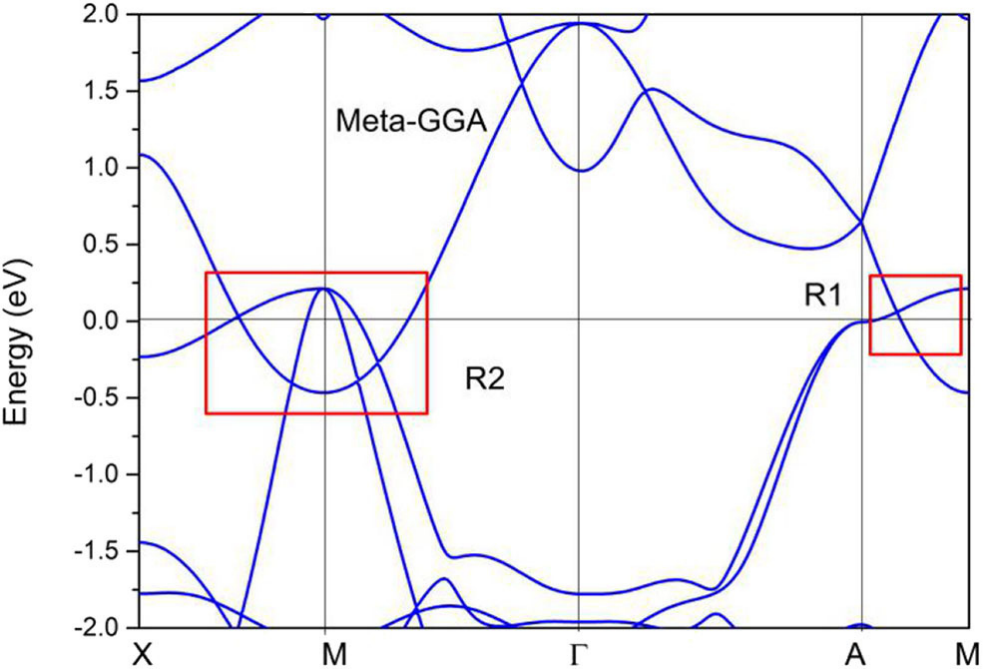
Calculated band structure of PtO along the X-M-Γ-A-M directions, as determined using the TPSS-meta-GGA method.

Without considering the spin–orbit coupling effect, the band structure of PtO at its equilibrium lattice constants along the Γ-X-M-Γ-Z-R-A-Z-A-M directions was calculated using the GGA method, and the result is given in Figure 7. PtO is a typical semimetal (Wang et al., 2020a,b; Yalameha and Nourbakhsh, 2020) with clean band crossing points, These band-crossing points are located around the Fermi level and far from other trivial bands. Interestingly, these band crossing points are concentrated mainly in the R1 and R2 regions. We will discuss each band crossing point in R1 and R2. To confirm the band crossing points near the Fermi level further, one type of meta-GGA method, Tao–Perdew–Staroverov–Scuseria (TPSS) (Sun et al., 2011), was selected to prove the occurrence of the band crossing points in the R1 and R2 regions. The PtO band structure along the X-M-Γ-A-M directions as determined via the TPSS-meta-GGA method is shown in Figure 8. Obviously, the band crossing points in both regions are retained.

**Figure 9:**
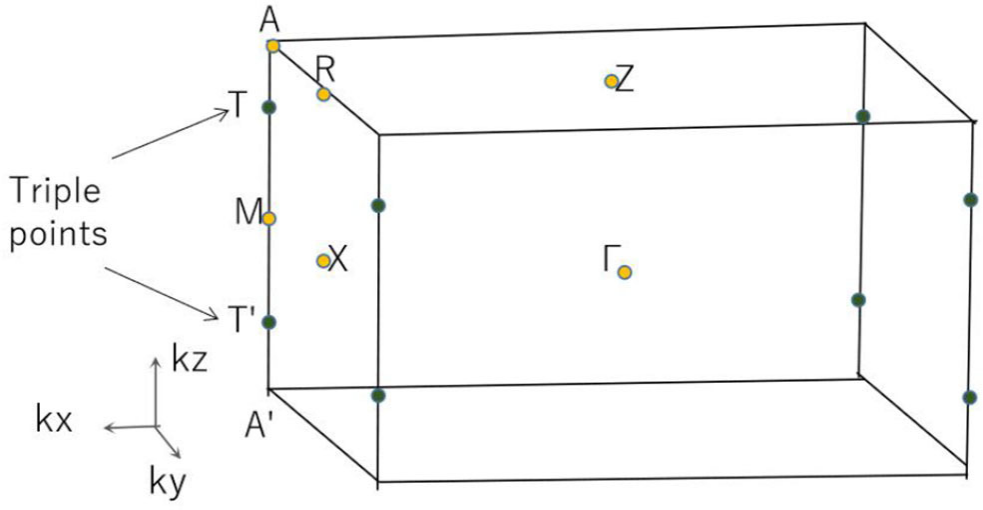
Triple nodal point positions (highlighted as green balls).

**Figure 10:**
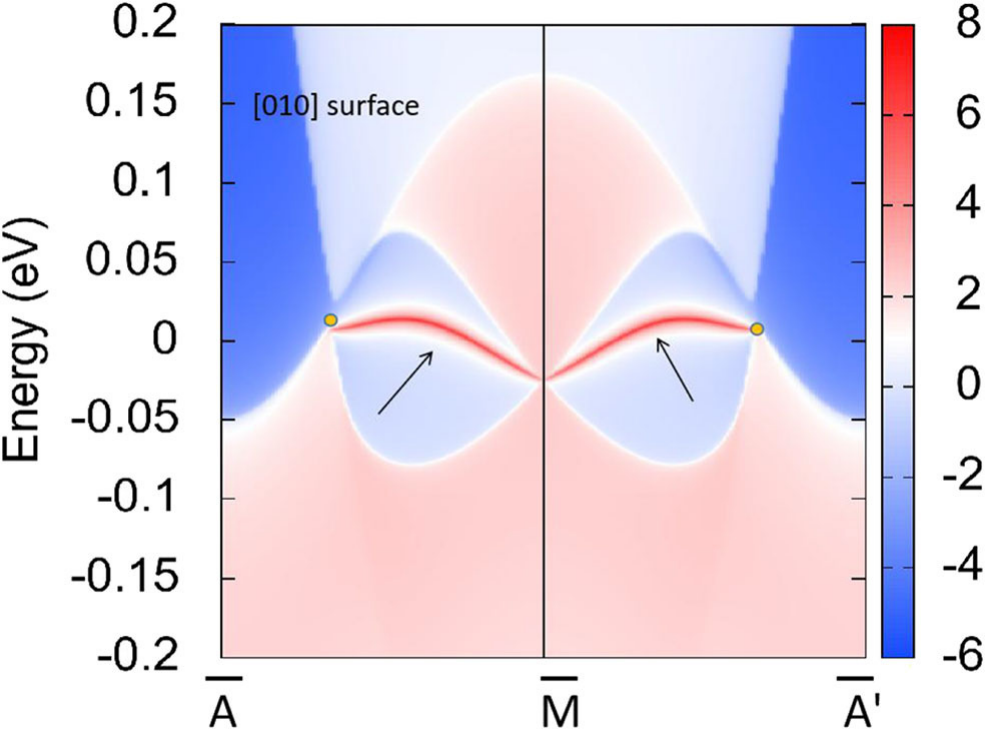
Projected spectrum on the (010) PtO tetragonal crystal surface. The band crossing points are marked as yellow balls and the Fermi arc surface states are highlighted using black arrows.

In R1, we can see that the band crossing point along the A-M direction induces a pair of triple nodal points. Symmetry analysis (with the help of Quantum ESPRESSO) shows that the two bands (conduction and valence bands), respectively, belong to irreducible representations Γ_2_ and Γ_2_ of the C_4v_ symmetry for the A-M path (in R1). This pair of triple nodal points is generated by one non-degenerate band and one doubly degenerate band. The triple nodal point locations are given in Figure 9. The Fermi arc surface state (Xu et al., 2015b; Jin et al., 2019b, 2020) can be seen as strong evidence for the appearance of triple nodal points. In Figure 10, we show the projected spectrum on the (010) PtO tetragonal crystal surface. The triple nodal points are highlighted as yellow balls and the obvious Fermi arc non-trivial surface states, which are located inside the band crossing points and marked by black arrows, are near the Fermi level.

**Figure 11:**
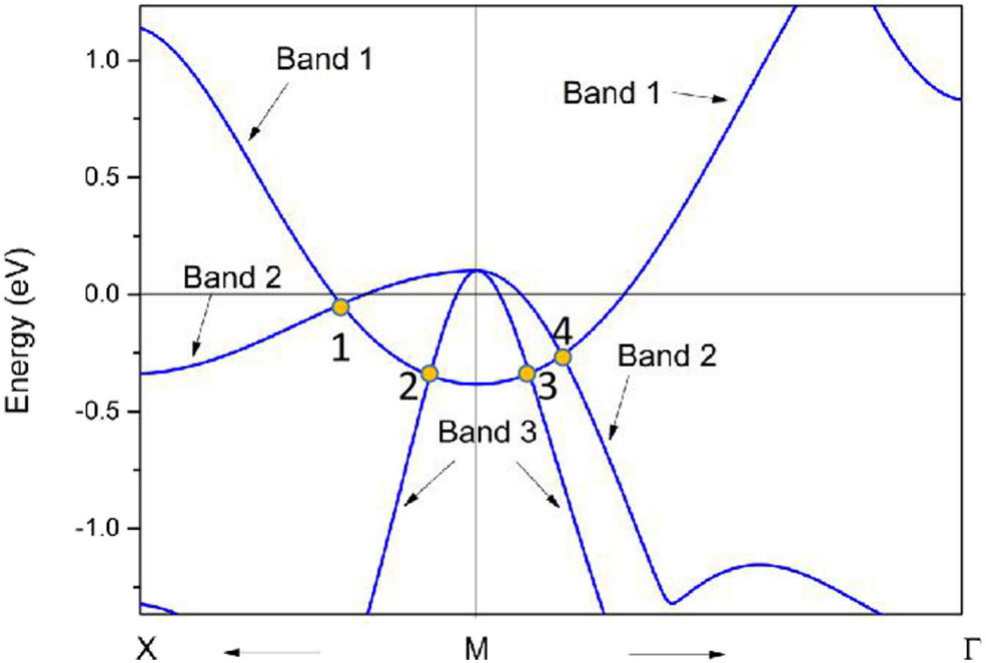
Calculated PtO band structure along the X-M-Γ directions, as determined using the GGA method. The band crossing points, labeled 1, 2, 3, and 4, are marked by yellow balls.

**Figure 12:**
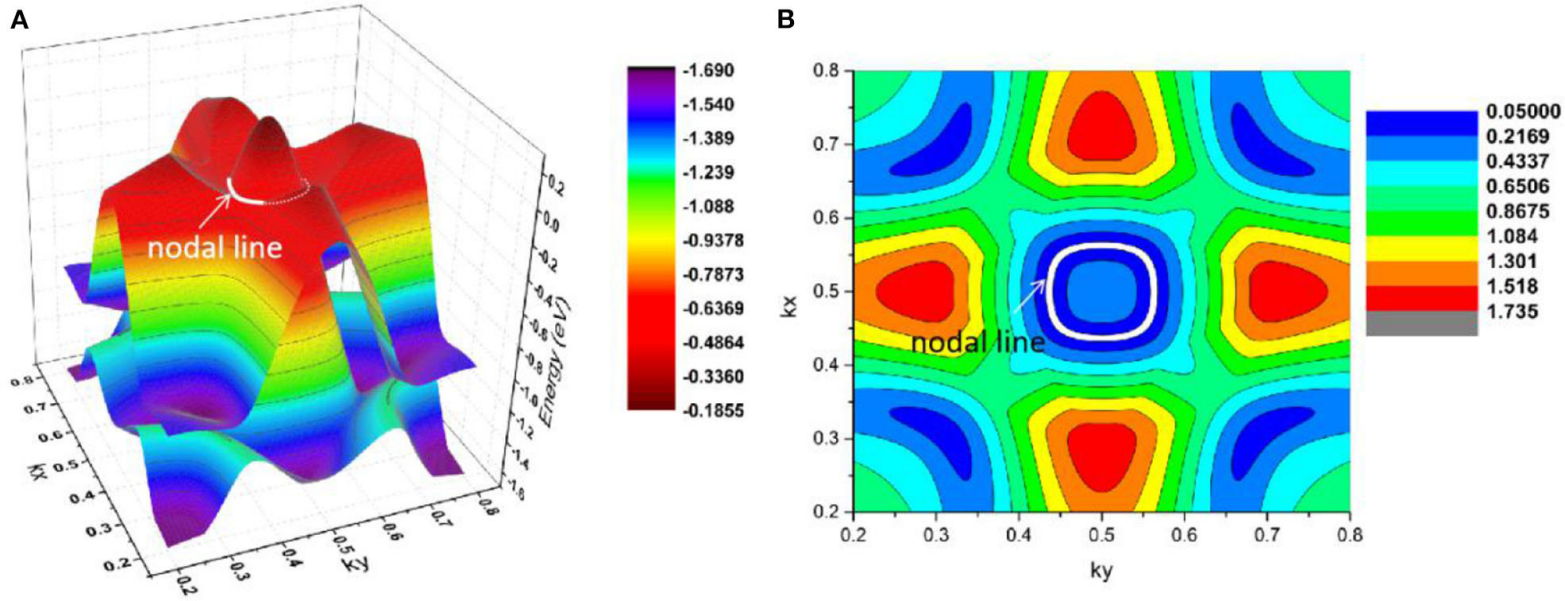
M-point-centered **(A)** 3-D and **(B)** 2-D plots of bands 1 and 3 in the *k*_*z*_ = 0 plane in R2. The white line indicates the nodal line in this plane.

**Figure 13:**
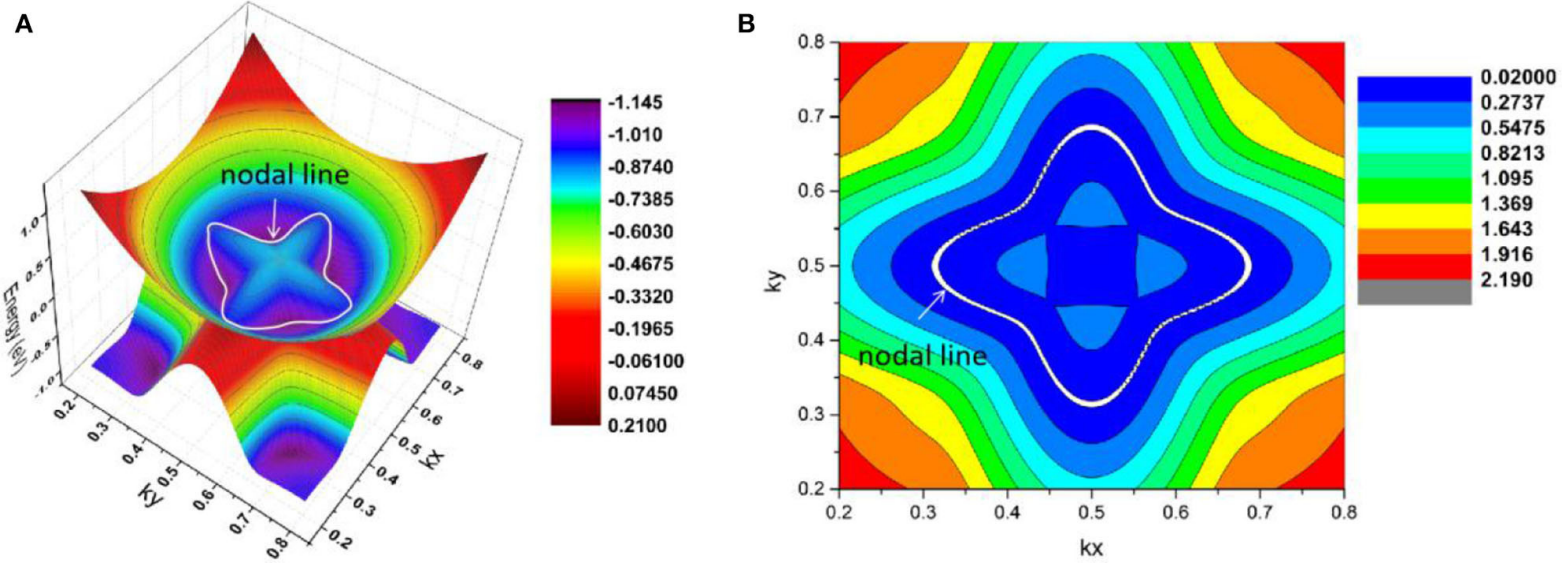
M-point-centered **(A)** 3-D and **(B)** 2-D plots of bands 1 and 2 in the *k*_*z*_ = 0 plane in R2. The nodal line is marked as a white line.

**Figure 14:**
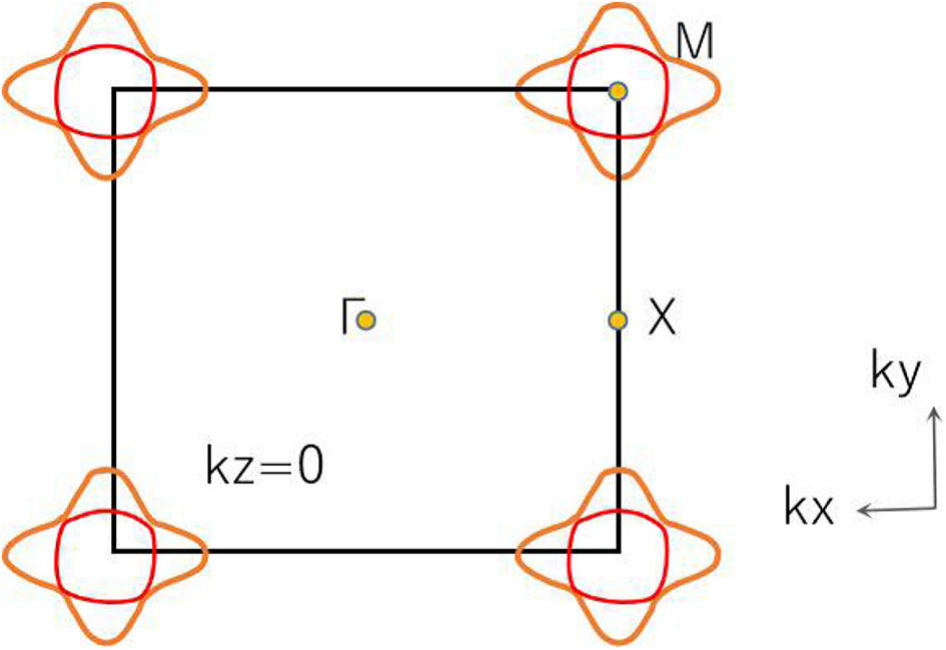
Illustration of M-point-centered double closed nodal lines in the *k*_*z*_ = 0 plane. Double nodal lines are marked using different colors.

As shown in Figure 11, there are four band crossing points along the X-M-Γ directions and near the Fermi level in R2. Band crossing points 1 and 4 are generated by crossing bands 1 and 2. However, band crossing points 2 and 3 are formed by overlaps between bands 1 and 3. Since PtO hosts both *P* and *T* symmetries, these four band crossing points along the X-M-Γ directions in the *k*_*z*_ = 0 plane cannot be treated as isolated points (Gao et al., 2018; He et al., 2019; Zhao et al., 2020). To further verify that these four band crossing points in R2 belong to nodal lines, 3-D and 2-D plots of bands 1 and 3 in the *k*_*z*_ = 0 plane are given in Figure 12. One M-point-centered closed nodal line, marked as a white line, occurs in the *k*_*z*_ = 0 plane. Similarly, the 3-D and 2-D plots of bands 1 and 2 in the *k*_*z*_ = 0 plane are shown in Figure 13. The other M-point-centered nodal line with a closed shape appears in the *k*_*z*_ = 0 plane. However, these two closed nodal lines exhibit different shapes and sizes, and are located at different energies. The closed nodal line shown in Figure 13 is larger than that in Figure 12. An overall illustration of the M-point-centered double closed nodal lines in the *k*_*z*_ = 0 plane is shown in Figure 14.

**Figure 15:**
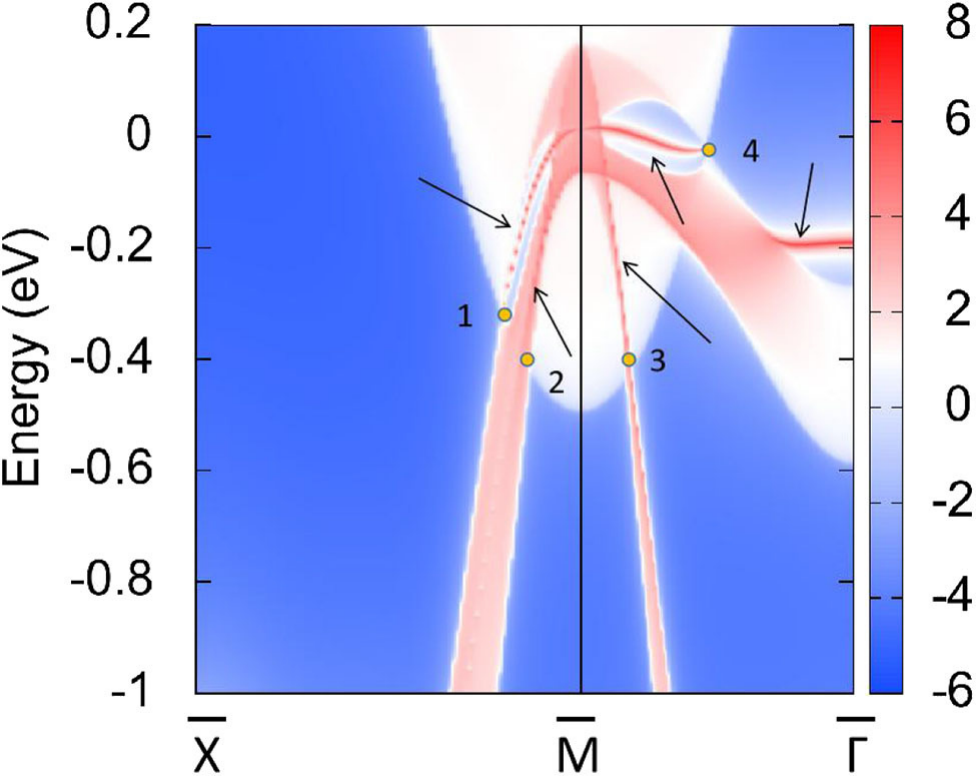
Projected spectrum on the (001) PtO tetragonal crystal surface. Band crossing points 1, 2, 3, and 4 are marked as yellow balls and the drum-head-like surface states are highlighted using black arrows.

Typically, drum-head-like surface states that originate from the bulk band crossing points can be observed (Zhou et al., 2018; Yi et al., 2019). To further prove this argument, the spectrum is projected on the (001) PtO tetragonal crystal surface in Figure 15. The drum-head-like surface states clearly appear outside and inside band crossing points 1, 2, 3, and 4.

## Conclusions

In summary, we have systematically used first-principles calculations to study the electronic, mechanical, and topological properties of tetragonal phase PtO, which is a realistic material. PtO is an excellent topological semimetal with pairs of triple nodal points and double closed nodal lines in the *k*_*z*_ = 0 plane when the spin–orbit coupling effect is ignored. The 0-D triple nodal points and 1-D closed nodal lines are further confirmed by the exotic Fermi arc surface states and drum-head-like surface states, respectively. The mechanical properties and phonon dispersion of this material allowed us to determine that PtO is mechanically stable, elastically ductile, and dynamically stable. These results demonstrate that PtO is an interesting material, which can be used to achieve experimental detection of nodal points and nodal lines or to further practical applications.

## Data Availability Statement

All datasets generated for this study are included in the article/Supplementary Material.

## Author Contributions

YL: software, supervision, and conceptualization. JX and VS: reviewing and editing. All authors contributed to the article and approved the submitted version.

## Conflict of Interest

The authors declare that the research was conducted in the absence of any commercial or financial relationships that could be construed as a potential conflict of interest.

## Footnotes

^1^Available online at: https://www.materialsproject.org/materials/mp-7947/.
