# Supplementary figures and images for: The Tetragonal Monoxide of Platinum: A New Platform for Investigating Nodal-Line and Nodal-Point Semimetallic Behavior
Source: Front Chem. 2020 Aug 14;8:704. doi: 10.3389/fchem.2020.00704 (PMC7457133; doi:10.3389/fchem.2020.00704)

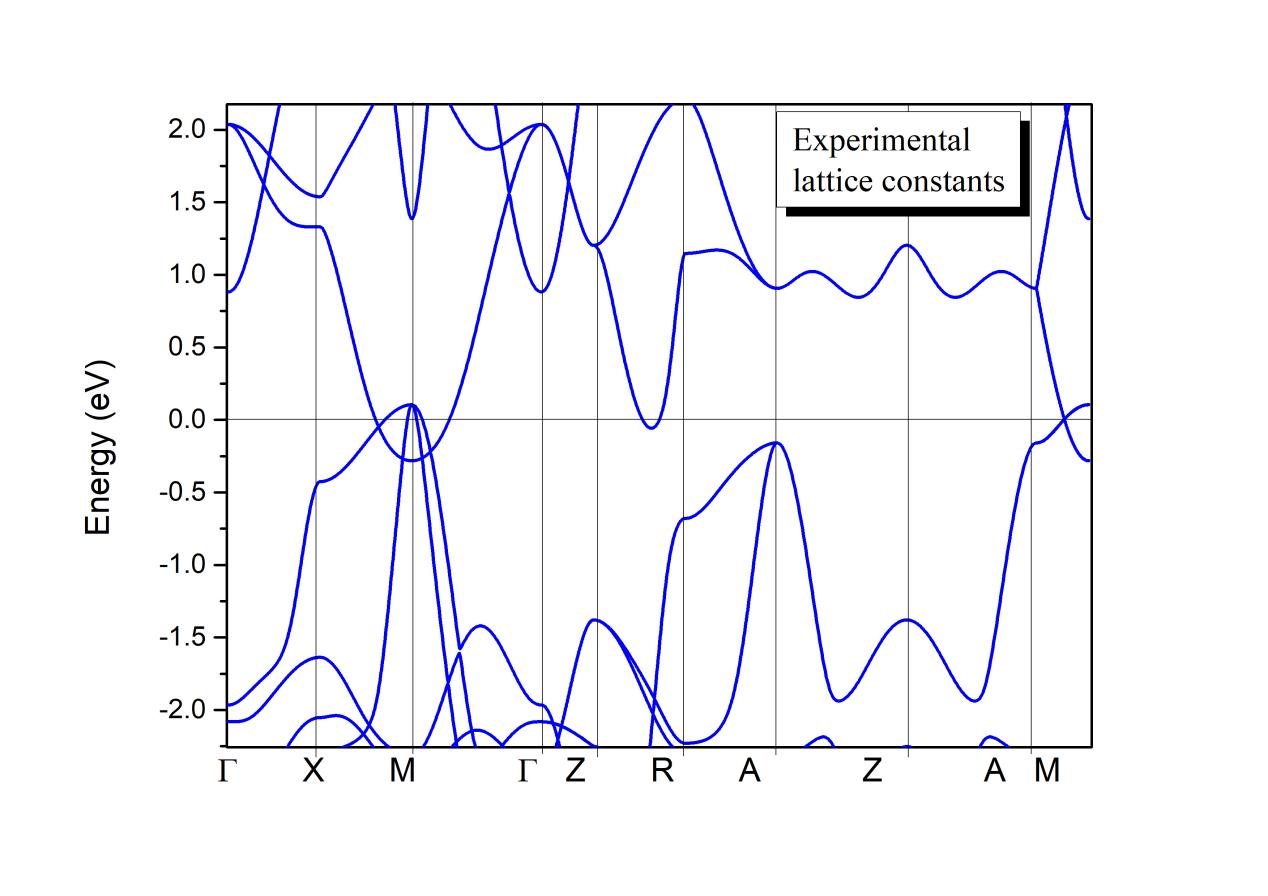


Figure S1. Band structure of PtO with experimental lattice constants.

Supplement: Supplementary file 1 [file Table_1.DOCX]
